# ACCEPT - combining acalabrutinib with rituximab, cyclophosphamide, doxorubicin, vincristine and prednisolone (R-CHOP) for Diffuse Large B-cell Lymphoma (DLBCL): study protocol for a Phase Ib/II open-label non-randomised clinical trial

**DOI:** 10.12688/f1000research.22318.1

**Published:** 2020-08-07

**Authors:** Andrew Davies, Sharon Barrans, Cathy Burton, Katy Mercer, Joshua Caddy, Fay Chinnery, Laura Day, Diana Fernando, Kirit Ardeshna, Graham Collins, John Radford, Simon Rule, Andrew McMillan, Peter Johnson, Gareth Griffiths

**Affiliations:** 1 Southampton Experimental Cancer Medicine Centre, University of Southampton and University Hospital Southampton NHS Foundation Trust, Southhampton, UK; 2 Leeds Teaching Hospitals NHS Trust, Leeds, UK; 3 Southampton Clinical Trials Unit, University of Southampton and University Hospital Southampton NHS Foundation Trust, Southampton, UK; 4 University College London Hospitals NHS Foundation Trust, London, UK; 5 Oxford University Hospitals NHS Trust, Oxford, UK; 6 University of Manchester, Manchester, UK; 7 Plymouth Hospitals NHS Trust and Plymouth University Peninsula School of Medicine and Dentistry, Plymouth, UK; 8 Nottingham University Hospitals NHS Trust, Nottingham, UK

**Keywords:** Diffuse large B-cell lymphoma, acalabrutinib, Btk inhibitor, R-CHOP, molecular profiling, phase I/II

## Abstract

**Background:** Over 13,000 new cases of non-Hodgkin’s lymphoma (NHL) are diagnosed in the UK, with approximately 4,900 attributable deaths each year. Diffuse Large B-cell Lymphoma (DLBCL) is the most common NHL comprising one third of adult NHL cases. R-CHOP (rituximab, cyclophosphamide, doxorubicin, vincristine, prednisolone) is accepted as the international standard first-line regimen, but improvement in first line treatment is needed. Dysregulated B-cell receptor (BCR) signalling has been identified as a feature of DLBCL. Inhibition of Bruton’s tyrosine kinase (Btk), downstream of the BCR has proven efficacious in other B-cell malignancies and in combination with R-CHOP. The second generation Btk inhibitor, acalabrutinib, may have improved target potency and specificity, and therefore better efficacy and tolerability.

**Methods: **ACCEPT is an open-label non-randomised Phase Ib/II trial testing the addition of acalabrutinib to conventional R-CHOP therapy. ACCEPT incorporates an initial 6+6 modified Phase I design of up to 24 participants followed by 15 participant single arm Phase II expansion cohort in treatment naive patients with histologically confirmed DLBCL expressing CD20. Participants are recruited from UK secondary care sites. Phase I will establish the recommended Phase II dose (RP2D, primary endpoint) of acalabrutinib in combination with R-CHOP. Phase II will gain additional information on safety and efficacy on the RP2D. The primary endpoints of Phase II are overall response rate and toxicity profile. Secondary endpoints include duration of response (progression-free survival and overall survival OS) in relation to cell of origin. Analyses are not powered for formal statistical comparisons; descriptive statistics will describe rates of toxicity, efficacy and translational endpoints.

**Discussion:** ACCEPT will provide evidence for whether acalabrutinib in combination with R-CHOP is safe and biologically effective prior to future Phase II/III trials in patients with previously untreated CD20 positive DLBCL.

**Trial registration: **EudraCT Number: 2015-003213-18 (issued 16 July 2015); ISRCTN 13626902 (registered 07 March 2017).

## Abbreviations

ABC, Activated B-cell; ADCC, Antibody dependent cellular cytotoxicity; AE, Adverse Event; AUC, Area under curve; BCR, B-cell Receptor; Btk, Bruton Tyrosine Kinase; CHOP, Cyclophosphamide, doxorubicin, vincristine, prednisolone; CLL, Chronic lymphocytic Leukaemia; Cmax, Maximum Concentration; COO, Cell of origin; CRF, Case Report Form; CT, Computer Tomography; CTCAE, Common Terminology Criteria for Adverse Events; DMEC, Data Monitoring and Ethics Committee; DLT, Dose Limiting Toxicity; DLBCL, Diffuse Large B-cell lymphoma; ECOG, Eastern Cooperative Group; FDA, Food and Drug Administration; FFPE, Formalin Fixed Paraffin Embedded; FISH, Fluorescence In Situ Hybridization; GCB, Germinal Centre B-cell; HBcAB, Hepatitis B core Antibody; HBsAB, Hepatitis B Surface antibody; HBsAg, Hepatitis B surface Antigen; HBV, Hepatitis B virus; HCV, Hepatitis C virus; HIV, Human Immunodeficiency Virus; HMDS, Haematological Malignancy Diagnostic Service; IMP, Investigational Medicinal Product; IV, Intravenous; LVEF, Left Ventricular Ejection Fraction; MAD, Maximal administered Dose; MHRA, Medicines and Healthcare products Regulatory Agency; MTD, Maximum Tolerated Dose; NCI, National Cancer Institute; NHL, Non-Hodgkin Lymphoma; Od, Once daily; ORR, Overall Response Rate; PBMC, Peripheral Blood Mononuclear cells; PK, Pharmacokinetic; PD, Progressive disease; PET CT, Positron Emission Tomography computer tomography; PIS, Patient Information Sheet; PO, By mouth; PR, Partial Response; RP2D, Recommended Phase 2 Dose; R-CHOP, Rituximab, cyclophosphamide, doxorubicin, vincristine, prednisolone; SAE, Serious Adverse Event; SCTU, Southampton Clinical Trial Unit; SUSAR, Suspected Unexpected Serious Adverse Reaction; Tmax, Time to maximum concentration

## Introduction

### Background and rationale

More than 13,000 new cases of non-Hodgkin’s lymphoma (NHL) were diagnosed in the UK between 2014–2016, with nearly 4,900 patients dying as a result of their disease in 2017 ^1^. Diffuse large B-cell lymphoma (DLBCL) is the most common type of NHL, accounting for 30–40% of NHL cases in adults with an annual incidence of about 5,500 patients in the UK ^1, 2^.

Cyclophosphamide, vincristine, doxorubicin and prednisolone (CHOP) used to be the standard of care for DLBCL. But, overall survival was disappointing; CHOP only cured about 30 percent of patients with advanced stages of intermediate-grade or high-grade NHL ^3^. Addition of the anti-CD20 monoclonal antibody rituximab to CHOP resulted in higher response rates, longer event free survival and improved overall survival. R-CHOP is now accepted as the international standard, with cure rates around 75% ^4– 8^.

Despite this improvement, many patients either fail to respond or relapse after having achieved an initial remission. Their prognosis is poor, because earlier rituximab therapy limits the success of salvage treatment and so the majority of these patients will die from their disease ^9^. Consequently, there is an urgent need to improve the effectiveness of first line treatment.

Gene expression profiling of untreated DLBCL samples has identified three distinct sub-classifications of the disease: activated B-cell like [ABC-DLBCL]; germinal centre B-cell like [GCB- DLBCL] and unclassified DLBCL. Each has its own biological features and clinical outcomes when treated with CHOP or R-CHOP.

The cells of ABC type are driven by “chronic active” B-cell receptor (BCR) signalling ^10^. The BCR signalling pathway can be disrupted using Bruton tyrosine kinase (Btk) inhibitors. Targeting BCR signalling by inhibiting Btk with ibrutinib (a first generation Btk inhibitor) in combination with R-CHOP is known to be safe for previously untreated B-cell NHL ^11^.

Acalabrutinib (Acerta Pharma B.V.) is a second generation Btk inhibitor with increased target selectivity compared to ibrutinib ^12^. As of 30 December 2018, acalabrutinib has been administered to over 2600 participants in clinical studies, including patients with haematologic malignancies, solid tumour, or rheumatoid arthritis, and participants who are healthy volunteers or with mild to moderate hepatic impairment. No serious adverse events (SAEs) have been reported in the hepatic impairment study or in the healthy volunteer studies. No expected SAEs have been identified for acalabrutinib to date.

We hypothesised that acalabrutinib would have improved target potency and specificity and is both active and tolerable in combination with R-CHOP.

## Objectives

The objectives of the study are described in Table 1. ACCEPT aims to establish a recommended Phase II dose (RP2D) for acalabrutinib when combined with R-CHOP.

**Table 1.** Primary, secondary and tertiary objectives of the ACCEPT study.

	Objective	Endpoint used to evaluate
**Primary:**	Phase I – Dose Escalation To propose a recommended dose for Phase II evaluation of acalabrutinib in combination with R-CHOP in patients with DLBCL: - To examine the safety and toxicity profile of acalabrutinib in combination with R-CHOP and defining the dose limiting toxicity or maximum administered dose.	Phase I Dose limiting toxicity of acalabrutinib combined to R-CHOP.
	Phase II: - Dose Expansion To document anti-tumour activity of acalabrutinib in combination with R-CHOP in patients with previously untreated CD20 positive DLBCL	Phase II Overall response rate of the combination acalabrutinib and R-CHOP.
	To determine additional safety information of acalabrutinib in combination with R-CHOP.	Safety of the combination acalabrutinib and R-CHOP.
**Secondary:**	To determine the pharmacokinetic (PK) profile of acalabrutinib when given in combination with R-CHOP in patients with DLBCL.	Pharmacokinetic of acalabrutinib, AUC, Cmax, Tmax, half-life T _1/2_ and other PK parameter.
	To evaluate the effect of acalabrutinib in combination with R-CHOP on outcomes according to COO	Overall response rate of the combination acalabrutinib and R-CHOP according to COO
	To measure the duration of response to acalabrutinib in combination with R-CHOP over a follow-up period of 2 years	2-years progression-free survival; 2-years overall survival.
**Tertiary:**	To determine BTK occupancy by acalabrutinib in peripheral blood mononuclear cells when given with R-CHOP.	Btk occupancy by acalabrutinib on peripheral blood using fluorescent affinity probe assay.
	To determine the impact of addition of acalabrutinib on R- CHOP mediated ADCC.	Antibody-dependent cell-mediated cytotoxicity of R-CHOP when combined to acalabrutinib, post 1st R-CHOP and at day 8, 2nd cycle acalabrutinib + R-CHOP.
	To determine evidence of B-cell receptor (BCR) activation in patients before and after treatment with combination of R-CHOP and acalabrutinib.	CD86 and CD69 expression as a function of BCR activation by flow cytometry.
	To explore the use of tumour-specific circulating DNA in plasma/serum as a non-invasive diagnostic and prognostic tool, with paired lymphoma tissue, through treatment of DLBCL and at follow up.	Tumour-specific DNA in plasma will be sequenced throughout treatment and compared with lymphoma tissue and clinical course.
	To explore correlation of molecular characteristics in tumour material to clinical outcomes.	Apply the following techniques to FFPE tumour material: mutational panel, FISH analysis, immunohistochemical analysis for dual protein expression of Myc and Bcl2 and gene expression profiling using whole transcriptome profiling.

Secondary objectives include the evaluation of dose limiting toxicity (DLT), assessed using CTCAE v4.03 at baseline, at each treatment cycle and at each follow-up visit; treatment compliance, assessed using patient diaries and returned Investigational Medicinal Product (IMP), as well as the electronic case report forms (eCRFs) during the treatment period; and response rate of participants enrolled in the Phase II part of the trial.

Translational objectives include determining the pharmacokinetic (PK) profile of acalabrutinib when given in combination with R-CHOP in patients with DLBCL. Btk occupancy by acalabrutinib of PBMC peripheral blood mononuclear cells (PBMCs) will be measured using fluorescent affinity probe assay. Pre- and post-dose blood samples will be used to measure the impact of the addition of acalabrutinib on R-CHOP mediated antibody dependent cellular cytotoxicity (ADCC), and measure BCR activation via CD86 and CD69 expression on PBMC by flow cytometry.

ACCEPT will prospectively validate the cell of origin (ABC versus GCB) model of DLBCL and its practicality and utility, as well as assessing the benefit/toxicity of the addition of acalabrutinib to R-CHOP. DNA extracted from tumour material will be used to perform mutation detection on BCR pathway (e.g. Btk, PI3K, CD79b). Genetic abnormalities of BCR pathway will be correlated with clinical outcomes and the expression of BCR pathway target genes.

### Trial design

**Figure 1.**
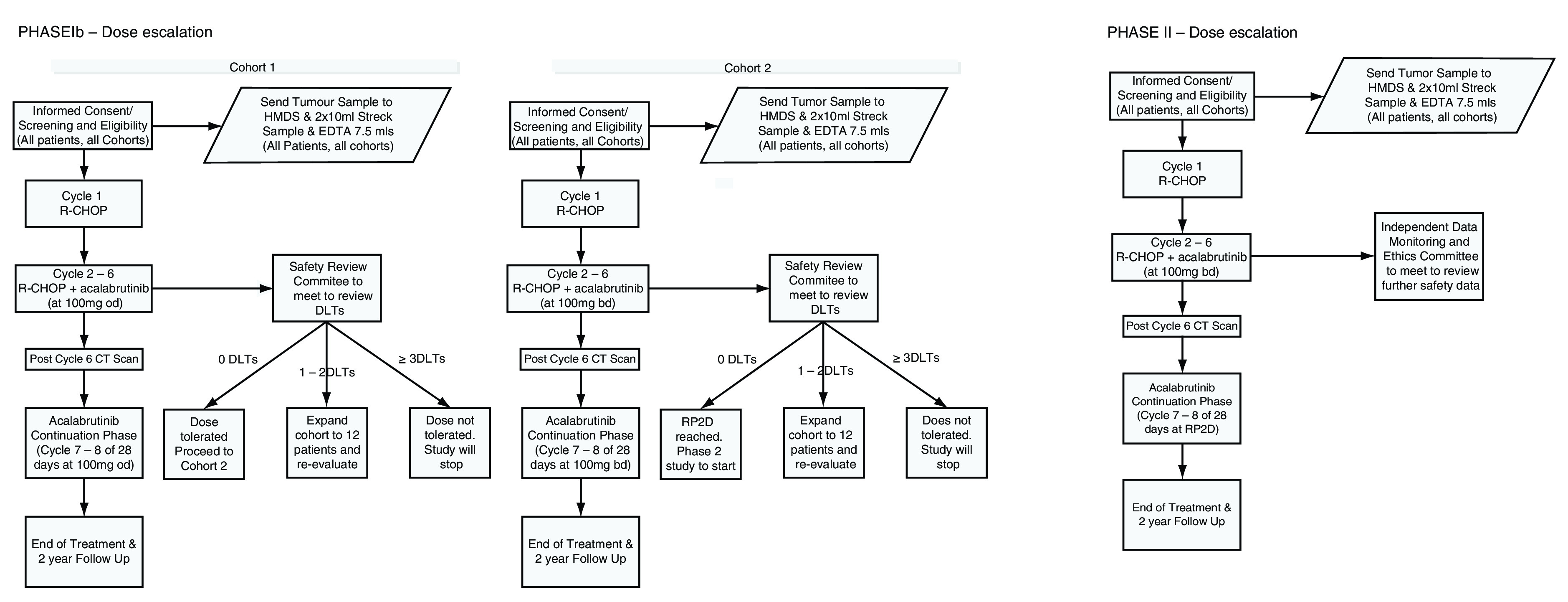
Accept Trial schema.

ACCEPT is a multicentre open-label non-randomised Phase Ib/II clinical trial conducted in two stages recruiting approximately 40 participants. Phase I will be dose escalation following the conventional rules of 6+6 modified design, escalation will proceed until a maximum tolerated dose (MTD) is defined or the maximal administered dose (MAD) is determined in order to define the RP2D. A six participant cohort design is employed to maximise patient safety ^13^, which will result in 6–24 participants recruited. Phase II will be an expansion cohort in order to gain additional information on safety and efficacy at the RP2D from a total of 15 participants recruited ( Figure 1).

## Methods: participants, interventions and outcomes

This is protocol V6 (15-Jul-2019).

### Study setting

Seven secondary care hospitals in the UK are recruiting participants into ACCEPT. A list of study sites is available via contacting accept@soton.ac.uk.

### Eligibility criteria

***Inclusion criteria***

Participants should fulfil the following criteria:

- Histologically confirmed DLBCL, expressing CD20; sufficient tumour block should be available to forward to a central laboratory for gene expression profiling and pathology review; at a minimum, participants should have sufficient tumour material to test for: H/E morphological check of compatibility of DLBCL diagnosis, immunophenotyping and RNA for gene expression profiling
- Measurable disease of at least 15mm
- Not previously treated for lymphoma and fit enough to receive combination chemoimmunotherapy with curative intent
- Stage IAX (bulk defined as lymph node diameter >10cm) to stage IV disease and deemed to require a full course of chemotherapy. Patients with non-bulky IE disease will not be eligible
- ECOG performance status 0–2 or 3 if this is directly attributable to lymphoma
- Adequate bone marrow function with platelets > 100×10 ^9^/L; neutrophils > 1.0×10 ^9^/L at study entry, unless lower figures are attributable to lymphoma
- Measured or calculated creatinine clearance > 30mls/min, (calculated using the formula of Cockcroft and Gault [(140-Age) × Mass (kg) × (1.04 (for women) or 1.23 (for men))/Serum Creatinine (µmolL)]
- Serum bilirubin < 35μmol/L and transaminases < 2.5× upper limit of normal at time of study entry
- Cardiac function sufficient to tolerate 300mg/m ^2^ of doxorubicin. A pre-treatment echocardiogram or MUGA is required to establish baseline LVEF equal to or greater than institutional normal range.
- No concurrent uncontrolled medical condition
- Life expectancy > 3 months
- Aged 16 years and above
- Willing and able to participate in all required evaluations and procedures in this study protocol including swallowing capsules without difficulty
- Ability to understand the purpose and risks of the study and provide signed and dated informed consent

***Exclusion criteria***

Patients will be excluded from the study entry if any of the following criteria are met:

- Previous history of treated or untreated indolent lymphoma; however newly diagnosed patients with DLBCL who are found to also have small cell infiltration of the bone marrow or other diagnostic material (discordant lymphoma) will be eligible
- Patients who have received immunisation with a live vaccine within four weeks prior to enrolment will be ineligible
- Diagnosis of primary mediastinal lymphoma
- Diagnosis of primary Central Nervous System lymphoma
- History of stroke or intracranial haemorrhage in preceding 6 months
- History of bleeding diathesis (e.g., haemophilia, von Willebrand disease)
- Requires or receiving anticoagulation with warfarin or equivalent antagonists (e.g. phenprocoumon) within 7 days of first dose of acalabrutinib; however, patients using therapeutic low molecule weight heparin or low dose aspirin will be eligible
- Prior exposure to a BCR inhibitor (e.g. Btk inhibitors, phosphoinositide-3 kinase (PI3K), or Syk inhibitors) or BCL-2 inhibitor (e.g. ABT-199)
- Requires treatment with a strong cytochrome P450 3A4 (CYP3A4) inhibitor/inducer.
- Requires treatment with proton pump inhibitors (e.g. omeprazole, esomeprazole, lansoprazole, dexlansoprazole, rabeprazole, or pantoprazole)
  ○ Patients receiving proton pump inhibitors should switch to short-acting H2-receptor antagonists or antacids prior to study entry to be eligible for enrolment into this study
- Uncontrolled systemic infection.
- Major surgery in the preceding 4 weeks of first dose of study drug. If a subject had major surgery, they must have recovered adequately from any toxicity and/or complications from the intervention before the first dose of study drug
- Significant cardiovascular disease such as uncontrolled or symptomatic arrhythmias, congestive heart failure, or myocardial infarction within 6 months of screening, or any Class 3 or 4 cardiac disease as defined by the New York Heart Association Functional Classification, or corrected QT interval (QTc) > 480 msec at screening. QTc interval should be calculated using Fridericia’s formula
- Serological positivity for Hepatitis B (HBV), C (HCV), or known HIV infection. As per standard of care, prior to initiation of immunochemotherapy, the results of hepatitis serology should be known prior to commencement of therapy.
  - Positive test results for chronic HBV infection (defined as positive HBsAg serology) will not be eligible. Patients with occult or prior HBV infection (defined as negative HBsAg and positive total HBcAb) will not be eligible. Patients who have protective titres of hepatitis B surface antibody (HBsAb) after vaccination will be eligible
  - Positive test results for HCV (HCV antibody serology testing) will not be eligible
- Women who can bear children must agree to use two highly effective forms of contraception or abstinence during the study and for 12 months after the last treatment dose (contraception is discussed under ‘5. Relevant concomitant care permitted or prohibited during the trial’)
- Breastfeeding or pregnant women
- Men who can father children must agree to use two highly effective forms of contraception with additional barrier or abstinence during the study and for 12 months after the last treatment dose (contraception is discussed under ‘Relevant concomitant care permitted or prohibited during the trial’)
- Men must agree to refrain from sperm donation during the study and for 12 months after the last treatment dose
- Serious medical or psychiatric illness likely to affect participation or that may compromise the ability to give informed consent
- Prior malignancy (other than DLBCL), except for adequately treated basal cell or squamous cell skin cancer, in situ cervical cancer, or other cancer from which the subject has been disease free for ≥ 2 years or which will not limit survival to < 2 years. Note: these cases must be discussed with Southampton Clinical Trials Unit (SCTU)
- Malabsorption syndrome, disease significantly affecting gastrointestinal function, resection of the stomach or small bowel, gastric bypass, symptomatic inflammatory bowel disease, or partial or complete bowel obstruction or gastric restrictions and bariatric surgery, such as gastric bypass
- Any immunotherapy within 4 weeks of 1 ^st^ dose of the study
- Concurrent participation in another therapeutic clinical trial

### Consent

***Who will take informed consent?***

Consent to enter the trial will be sought from each participant only after a full explanation has been given, a Patient Information Sheet (PIS) offered ( *Extended data* ^14^) and time allowed for consideration. Signed participant consent will be obtained. Only site staff named on the Delegation Log and authorised to do so may obtain consent. Patients may refuse to participate without giving reasons and this will not prejudice their future treatment.

***Additional consent provisions for collection and use of participant data and biological specimens***

The trial’s Informed Consent Forms detail the consent provisions for collection and use of participant data and biological specimens in future research ( *Extended data* ^14^).

### Interventions

***Explanation for the choice of comparators***

*Rationale for combination of acalabrutinib in combination with R-CHOP in DLBCL*

Dysregulation of BCR signalling is well recognised in DLBCL and other B-cell malignancies. Btk is central to signalling through the BCR. The safety and efficacy of a first generation Btk inhibitor, ibrutinib, in combination with R-CHOP has already been investigated by a Phase Ib study in patients with DLBCL, demonstrating that it is well tolerated with an overall response rate (ORR) of 91% (CR 70%) ^10^.

Acalabrutinib is a second generation Btk inhibitor with enhanced kinase selectivity and potential for better efficacy and tolerability over first-generation inhibitors. It has a number of preclinical properties that would indicate the potential for favourable efficacy and safety over ibrutinib. These include preferential selectivity for Btk with the potential for less off-target effects (e.g. Epidermal Growth Factor Receptor and diarrhoea). Acalabrutinib does not appear to abrogate thrombus formation, compared to ibrutinib, mediated by off-target kinase activity, and would therefore potentially reduce the risk of bleeding and also compared to ibrutinib does not have a negative effect on ADCC, an important mediator of rituximab function.

Results from the study of acalabrutinib in patients with chronic lymphocytic leukaemia (CLL) demonstrate a favourable toxicity profile with an emergent lower rate of haematological toxicity compared to ibrutinib. No DLTs were reached and no SAEs reported to date at doses ≤400mg ^15^.

*Rationale for dose selection*

Results from the study of ibrutinib in combination with R-CHOP indicated that the maximal tolerated dose was not reached and the recommended Phase II dose of ibrutinib was 560mg od ^10^. This is the licensed single agent dose for use in mantle cell lymphoma. Given the favourable single agent toxicity profile of acalabrutinib in the CLL study compared to ibrutinib, it is anticipated that acalabrutinib may safely be given in combination with R-CHOP at doses close or equivalent to those being investigated as a single agent.

Preliminary data from the ongoing Phase I/II study in patients with relapsed/refractory or previously untreated CLL have shown that acalabrutinib is well tolerated at dosages of 100 to 400 mg od and 100 to 200 mg bd ^15^. These data suggest that *de novo* synthesis of Btk can occur within 24 hours in peripheral blood cells. Twice daily dosing may ensure Btk inhibition for the entire 24 hours and thus may be beneficial in terms of increasing efficacy and/or decreasing development of resistance to acalabrutinib.

Taken together, the proposed starting dose of 100mg od is considered represent one where there is good pharmacodynamic evidence that the target is being suitably occupied without safety concerns in single agent studies and one supported by our pharmacokinetic knowledge of acalabrutinib. Escalation to twice daily dosing in the second cohort, addresses the continued inhibition of Btk based upon concerns about *de novo* synthesis during the 24-hour period.

***Intervention description***

All participants will receive 6 cycles of R-CHOP on a standard 21 day schedule with the addition of acalabrutinib in cycles 2–6. This will be followed by a continuation period of acalabrutinib only for 2 cycles each of 28 days. The Schedule of Events ( Table 2) describes the trial interventions.

**Table 2.** Schedule of observations and procedures.

R egistration and C ycle 1												
Visit	Screening ^a^											Cycle 1
Weeks												1
Days	Within 90 days of treatment		Within 35 days of treatment		Within 28 days of treatment		Within 14 days of treatment		Within 72 hours of treatment		Prior to treatment	R-CHOP
Informed consent					X							
Inclusion/Exclusion criteria							X					
Medical History							X					
Physical Exam							X					
IPI and NCCN-IPI ( *Extended data*)							X					
Vital signs ^b^							X					X
ECOG performance Status							X					X
PET-CT or contrast enhanced CT with separate PET ^c^			X									
Bone marrow biopsy ^d^	X											
Biochemistry: renal and liver function ^e^							X					
Biochemistry: Additional baseline panel ^f^									X			
Haematology ^g^									X			
Immunoglobulins									X			
Hepatitis B, C and HIV serology ^h^					X							
Pregnancy test ^i^											X	
Electrocardiogram ^j^					X							
Echocardiogram/ MUGA ^k^					X							
Cerebrospinal fluid examination ^l^ ^(if clinically indicated)^					X							
Tumour material ^m^					X							
Streck Plasma DNA Sample ^n^					X							
EDTA Sample ^n^											X	
Concomitant Medications							X					X
Adverse Events ^o^					X		X		X		X	X
R-CHOP												X
**C ycles 2 – E nd of T reatment**												
Visit	Cycle 2 Day 1	Cycle 2 Day 8	Cycle 2 Day 15	Cycle 3 Day 1	Cycle 4 Day1	Cycle 4 Day 8	Cycle 5 Day 1	Cycle 6 Day 1	Cycle 7 ^x^	Cycle 8 ^x^	End of Treatment ^bb^	
Weeks	4	5	6	7	10	11	13	16	19 ^w^	23	27–30	
Physical Exam ^y^	X			X	X		X	X	X	X	X	
Vital Signs ^p^	X	X	X	X	X	X	X	X	X	X	X	
ECOG performance Status ^y^	X	X	X	X	X	X	X	X	X	X	X	
Contrast enhanced CT									X ^aa^			
PET-CT or contrast enhanced CT and separate PET ^q^											X ^q^	
Bone marrow biopsy ^r^											(X) ^r^	
Biochemistry: renal and liver function ^s^	X	X	X	X	X	X	X	X	X	X	X	
Haematology ^t^	X	X	X	X	X	X	X	X	X	X	X	
Electrocardiogram ^z^				X			X		X		X	
Echocardiogram ^z^					X						X	
Pharmacokinetic samples ^cc^	X	X	X	X								
Pharmacodynamic samples ^cc^	X	X			X							
R-CHOP + acalabrutinib	X			X	X		X	X				
Acalabrutinib (only) ^x^									X	X		
Compliance ^u^	X	X	X	X	X	X	X	X	X	X	X	
Concomitant medications	X	X	X	X	X	X	X	X	X	X	X	
Adverse Events	X	X	X	X	X	X	X	X	X	X	X	
Streck Plasma DNA Sample ^v^	X			X							X	
Immunoglobulins				X							X	
Pregnancy Test	X			X	X		X	X	X	X	X	
**S chedule of F ollow-U p V isits** ( begin 3 months following cycle 8 - at the end of the acalabrutinib continuation phase- at week 39)												
Months following last therapy ^gg^	3		6	9		12	16	20		24		
Physical Exam	X		X	X		X	X	X		X		
ECOG performance status	X		X	X		X	X	X		X		
Haematology	X		X	X		X	X	X		X		
Biochemistry ^dd^	X		X	X		X	X	X		X		
CT ^ee^						X				X		
Adverse Events	X		X	X		X	X	X		X		
Streck Plasma DNA Sample ^ff^	X		X	X		X	X	X		X		
Immunoglobulins			X			X				X		
Pregnancy Test	X		X	X		X						

^a^ Screening investigations to be performed within 14 days of starting study medication with the exception of informed consent, CT, Electrocardiogram and Bone marrow biopsy ^b^ Blood pressure, pulse, temperature, height and weight. Assessment to be performed pre-dose and as per local practice during rituximab infusion ^c^ PET and Contrast Enhanced CT of chest, abdomen and pelvis (neck if indicated) should be carried out within 35 days of planned treatment. The PET-CT hybrid scanners may be used to acquire the required CT images only if the CT produced by the scanner is of diagnostic quality and includes the use of intravenous (IV) contrast. If this cannot be achieved, a PET and separate Contrast Enhanced CT scan should be performed. Bi-dimensional measurements are expected. ^d^ Bone marrow aspirate and trephine biopsy (single site with adequate trephine) (within 90 days of first treatment). ^e^ Serum chemistry to include sodium, potassium, urea, creatinine, bilirubin, alanine or aspartate transaminase, alkaline phosphatase and albumin. ^f^ Additional serum chemistry to be performed only at baseline: LDH, calcium, phosphate, β2-microglobulin and uric acid. ^g^ Full blood count to include haemoglobin, white blood cell count, absolute neutrophil, lymphocytes and platelets counts. To be taken within 72 hours of chemotherapy administration on each cycle ^h^ As per standard of care, sites should have hepatitis results available prior to initiation of immunochemotherapy. ^i^ Only required in females of child bearing potential on day 1 before treatment commences ^j^ A 12 lead ECG should be performed on all patients. ^k^ In addition, an Echocardiogram or MUGA will be performed for all patients to establish a left ventricular ejection fraction equal to or greater than institutional normal range. Patients should be considered suitable to receive 300mg/m ^2^ doxorubicin ^l^ Cerebrospinal fluid examination should be performed if clinically indicated or lymphomatous involvement of peripheral blood, nasal/paranasal sinuses or testis. CNS prophylaxis may be given according to local policy ^m^ Diagnostic tumour block to be forwarded immediately upon obtaining either Tissue Block Screening consent or main study consent (for those patients whose tissue sample is easily accessible) to HMDS, Leeds, according to study procedure outlined in Investigator Site File. ^n^ 20ml Blood sample in 2x10ml Streck tubes and 1x7.5ml EDTA Blood sample to be forwarded immediately to HMDS, Leeds prior to treatment start. ^o^ Adverse Events to be collected from date of consent. Only Adverse Events related to study procedures should be reported prior to start of treatment. ^p^ Blood pressure, pulse and temperature. Assessment to be performed pre-dose and as per local practice during rituximab infusion ^q^ PET and Contrast Enhanced CT of chest, abdomen and pelvis (neck if indicated) needs to be completed within 3 weeks of the patient completing last treatment cycle. The PET-CT hybrid scanners may be used to acquire the required CT images only if the CT produced by the scanner is of diagnostic quality and includes the use of intravenous (IV) contrast. If this cannot be achieved, a PET and separate Contrast Enhanced CT scan should be performed. Bi-dimensional measurements are expected. ^r^ Bone marrow biopsy to be repeated at the end of treatment if initially involved (to confirm CR) ^s^ Serum chemistry to include sodium, potassium, urea, creatinine, bilirubin, alanine or aspartate transaminase, alkaline phosphatase, LDH and albumin. To be taken within 72 hours of administration of each cycle ^t^ Full blood count including haemoglobin, white cell count, absolute neutrophil count, lymphocytes and platelets. To be taken within 72 hours of chemotherapy administration on each cycle ^u^ Compliance will be assessed by patient’s diary with date and time of acalabrutinib administration, and the count of remaining capsules ^v^ 20ml Blood sample in 2x10ml Streck tubes to be forwarded immediately to HMDS, Leeds ^w^ End of RCHOP + acalabrutinib treatment at week 19 is also the start of the continuation phase of acalabrutinib. The dosing of acalabrutinib is continued after the last day of treatment (cycle 6 day 21) for 56 days further. ^x^ Acalabrutinib only is given for cycles 7 and 8 as a continuation phase on a 28 days per cycle schedule - a total of 56 days continuation phase. ^y^ Physical exam and ECOG performance status. To be taken within 48 hours of acalabrutinib administration. ^z^ Electrocardiogram and Echocardiogram to be taken within 48 hours of acalabrutinib administration. ^aa^CT scan to be completed during Week 19 (+/- 1 week) ^bb.^ EoT visit should take place within 3 weeks from the final dose of acalabrutinib. ^cc^ Post dose samples should be taken at the protocol specified time ± 5 minutes. ^dd^ Serum chemistry to include sodium, potassium, urea, creatinine, bilirubin, alanine or aspartate transaminase, alkaline phosphatase, LDH and albumin. ^**ee**^ A contrast enhanced CT scan of the neck, chest, abdomen and pelvis will be performed at 12 months and 24 months following completion of protocol specified therapy. Bi-dimensional measurements are expected. ^ff^ 20 ml Blood sample in 2x10ml Streck tubes to be forwarded immediately to HMDS, Leeds. ^gg^ A visit window of +/- 2 weeks is permitted for follow-up visits. NB: The Participant/legal representative is free to withdraw consent at any time without providing a reason. When withdrawn, the participant will continue to receive standard clinical care. Follow up data will continue to be collected (unless the participant/legal representative has specifically stated that they do not want this to happen.

*Phase I - Dose Escalation*

*Stage 1:*

R-CHOP + Acalabrutinib (Cycle 2–6)

- Rituximab 375mg/m ^2^ IV, on day 1
- Cyclophosphamide 750mg/m ^2^ IV, on day 1
- Doxorubicin 50mg/m ^2^ IV, on day 1
- Vincristine 1.4mg/m ^2^ (max 2mg) IV, on day 1
- Prednisolone 100mg po, on day 1–5
- Acalabrutinib, 100mg once daily taken orally, days 1–21

Cycle 7 (28 days) and 8 (28 days) - Acalabrutinib only

- Acalabrutinib, 100mg once daily taken orally for 56 days

Acalabrutinib dosage:

The first six participants enrolled in the study (cohort 1) will receive 100mg od started on day 1 of the 2 ^nd^ cycle of R-CHOP. When participants of the first cohort have completed their third cycle, and their safety data has been reviewed by the Safety Review Committee, the decision for dose escalation to 100mg bd for cohort 2 could be taken.

*Stage 2:*

R-CHOP + Acalabrutinib (Cycle 2–6)

- Rituximab 375mg/m ^2^ IV, on day 1
- Cyclophosphamide 750mg/m ^2^ IV, on day 1
- Doxorubicin 50mg/m ^2^ IV, on day 1
- Vincristine 1.4mg/m ^2^ (max 2mg) IV, on day 1
- Prednisolone 100mg po, on day1–5
- Acalabrutinib 100mg twice daily, orally days 1–21

Cycle 7 (28 days) and 8 (28 days) - Acalabrutinib only

- Acalabrutinib, 100mg twice daily taken orally for 56 days following end of treatment with 6 cycles R-CHOP.

*Phase II – Dose Expansion*

R-CHOP + Acalabrutinib (Cycle 2–6)

- Rituximab 375mg/m ^2^ IV, on day 1
- Cyclophosphamide 750mg/m ^2^ IV, on day 1
- Doxorubicin 50mg/m ^2^ IV, on day 1
- Vincristine 1.4mg/m ^2^ (max 2mg) IV, on day 1
- Prednisolone 100mg po, on day 1–5
- Acalabrutinib at a Recommended Phase II dose

Cycle 7 (28 days) and 8 (28 days) - Acalabrutinib only

Acalabrutinib, at RP2D taken orally for 56 days following end of treatment with 6 cycles R-CHOP.

***Criteria for discontinuing or modifying allocated interventions***

After the participant has entered the trial, the clinician remains free to give alternative treatment to that specified in the protocol at any stage if he/she feels it is in the participant’s best interest, but the reasons for doing so should be recorded. In these cases, participants will be withdrawn from protocol treatment but remain within the trial for the purposes of follow-up and data analysis. All participants are free to withdraw at any time from the protocol treatment without giving reasons and without prejudicing further treatment.

***Strategies to improve adherence to interventions***

The investigator and/or study personnel will assess participant compliance with acalabrutinib at each study visit using direct questioning, examination of participants’ drug administration diaries and pill counts.

### Relevant concomitant care permitted or prohibited during the trial

***Supportive care***

- Live vaccination whilst on rituximab is prohibited and for participants who have peripheral B cell depletion must not have a live vaccination within three months of last rituximab dose
- Participants will receive standard anti-emetic as per local policy
- Allopurinol 300mg orally od should be given as per local practice; rasburicase may be administered if high risk of tumour lysis syndrome
- Mouth care therapies are given according to local policy
- Antacids and calcium supplements should be avoided for a period of at least 2 hours before and after taking acalabrutinib; short acting H2 receptor antagonists should only be taken at least 2 hours following acalabrutinib administration
- Diarrhoea and constipation are both recognised side effects of therapy in this protocol, diarrhoea is the most common; treatment should be given according to local policy, although prophylactic anti-diarrhoeal agents are not recommended
- Granulocyte-colony stimulating factor (GCSF) is mandated for all participants receiving R-CHOP and acalabrutinib; the formulation and duration will be according to local policy for primary prophylaxis but should be for at least seven days or until neutrophil recovery for those participants receiving the non-pegylated formulation
- Antimicrobial prophylaxis given against *Pneumocystis jirovecii* pneumonia is mandated
- Suitable infective prophylaxis to be given to participant’s aged 65 and over as per local policy; ciprofloxacin prophylaxis should be avoided due to possible interactions with acalabrutinib

***Prohibited therapies***

Acalabrutinib is not a strong direct inhibitor or inducer or CYP isoforms; thus, acalabrutinib, at the currently used clinical doses, is unlikely to be a perpetrator of a drug interaction at the level of inhibition or induction of CYP isoforms. Acalabrutinib is partially metabolised by CYP3A; its exposure is affected when co-administered with strong CYP3A4 inducers or inhibitors. Consequently, the concomitant use of strong inhibitors/inducers of CYP3A4 (see Table 3) should be avoided when possible.

**Table 3.** Known strong *in vivo* inhibitors and inducers of CYP3A4.

Strong Inhibitors of CYP3A ^a^	Strong Inducers of CYP3A ^e^
boceprevir clarithromycin ^b^ conivaptin ^b^ grapefruit juice ^c^ itraconazole ^b^ ketoconazole ^b^ indinavir lopinavir/ritonavirb (combination drug) mibefradild nefazodone nelfinavir posaconazole ritonavirb saquinavir telaprevir telithromycin voriconazole	carbamazepine ^f^ phenytoin ^f^ rifampin ^f^ St John's wort ^f^

a. A strong inhibitor for CYP3A is defined as an inhibitor that increases the AUC of a substrate for CYP3A by ≥ 5-fold.

b. In vivo inhibitor of P-glycoprotein.

c. The effect of grapefruit juice varies widely among brands and is concentration-, dose-, and preparation dependent. Studies have shown that it can be classified as a “strong CYP3A inhibitor” when a certain preparation was used (eg, high dose, double strength) or as a “moderate CYP3A inhibitor” when another preparation was used (eg, low dose, single strength).

d. Withdrawn from the United States market because of safety reasons.

e. A strong inducer for CYP3A is defined as an inducer that results in ≥ 80% decrease in the AUC of a substrate for CYP3A.

f. In vivo inducer of P-glycoprotein.

Note: The list of drugs in these tables is not exhaustive. Any questions about drugs not on this list should be addressed to the SCTU Source: FDA Drug Development and Drug Interactions: Table of Substrates, Inhibitors and Inducers. Web link Accessed 21 January 2015: http://www.fda.gov/Drugs/DevelopmentApprovalProcess/DevelopmentResources/DrugInteractionsLabeling/ucm093664.htm#inVivo

Based on these considerations, patients who require therapy with drugs listed in Table 3 should not be enrolled into the study. If medically justified, patients may be enrolled if such inhibitors or inducers can be discontinued or alternative drugs that do not affect these enzymes can be substituted within 7 days before first dose of study drug. If a subject requires a strong CYP3A4 while on study, the subject will be monitored closely for potential drug-related toxicities.

The effect of agents that reduce gastric acidity (e.g., proton pump inhibitors, H2 receptor antagonists or antacids) on acalabrutinib absorption was evaluated in a healthy volunteer study (ACE-HV-004; Acalabrutinib Investigator Brochure v8 ACERTA PHARMA B.V.). Results from this study indicate that participants should avoid the use of calcium carbonate containing drugs or supplements (e.g., antacids and calcium supplements) for a period of at least 2 hours before and after taking acalabrutinib. Similarly, participants should avoid the use of H2-receptor antagonists for a period of 2 hours after taking the study drugs. Use of omeprazole, esomeprazole, lansoprazole or any other proton pump inhibitors while taking acalabrutinib is not recommended due to a potential decrease in study drug exposure. However, if a subject requires the use of a proton pump inhibitor while on study (e.g., to treat a gastric ulcer) treatment options will be discussed with SCTU.

In circumstances where treatment with ciprofloxacin is needed, the dose of acalabrutinib should be reduced to 100mg od.

Warfarin and equivalent Vitamin K antagonists are prohibited. However, participants may use therapeutic low molecule weight heparin or low dose aspirin.

***Dietary restrictions***

Acalabrutinib can be taken with or without food. Because acalabrutinib may be metabolized by CYP3A4, participants should be strongly cautioned against consumption of grapefruit, grapefruit juice, or Seville orange juice (which contain potent CYP3A4 inhibitors) or using herbal remedies or dietary supplements (in particular, St John’s Wort, which is a potent CYP3A4 inducer). Otherwise, participants should maintain a normal diet unless modifications are required to manage an AE such as diarrhoea, nausea or vomiting.

***Contraception***

Contraception is mandated from trial entry until 12 months after the last treatment dose for participants of reproductive potential.

Participants with reproductive potential must use two highly effective methods of contraception during the study, with male participants also agreeing to use additional barrier or abstinence due to the risk of exposure to a developing foetus if the female partner is already pregnant.

Hormonal contraception may be susceptible to interaction with study or other drugs, which may reduce the efficacy of the contraception method.

Both male and female patients must be counselled about future fertility prospects and sperm/ovarian preservation prior to study entry. Male patients must refrain from donating sperm once on study and during treatment and the 12 month follow-up period. Acalabrutinib is not known to confer long term reduction in fertility, however the cytotoxic drugs used in this protocol, i.e. doxorubicin and cyclophosphamide, may do.

***Provisions for post-trial care***

Participants completing the trial, or who withdraw their consent for follow-up, will be managed by standard clinical care from their usual treating clinician.

### Outcomes

***Primary outcomes***

The Phase I study will be dose escalation that will proceed until an MTD is defined or the MAD is determined in order to define the RP2D of acalabrutinib in combined with R-CHOP. This dose will be used in the Phase II evaluation of acalabrutinib in combination with R-CHOP in participants with DLBCL.

The DLT will be defined using the National Cancer Institute (NCI) Common Terminology Criteria for Adverse events (CTCAE) version 4.03. The MTD is defined as the highest dose level at which none of the first six treated participants, or no more than 2 of the first 12 treated participants, experiences a DLT.

The DLT reporting period is from the start of treatment until the end of cycle 3. Events meeting the below criteria following the end of cycle 3 will not be recorded as DLTs.

A DLT will be defined as the occurrence of the following:

- Grade 2 or greater haemorrhagic event requiring medical intervention or any intracranial haemorrhage
  - Grade 3 or greater non-haematological toxicity at least possibly related to acalabrutinib (including grade 3 or 4 biochemical AEs). The following will be excluded; Grade 3 or 4 nausea in participants who have not received optimal treatment with anti-emetics, Grade 3 or 4 diarrhoea in participants who have not received optimal treatment with anti-diarrhoeal therapy, alopecia and those participants experiencing grade 3 or 4 administration reactions from rituximab.
- Grade 4 thrombocytopenia or neutropenia for more than 7 days despite GCSF use.
- Any complete, continuous dose interruption more than 7 days for acalabrutinib related toxicities of grade 2 or greater within cycle 2.

If a DLT attributed to acalabrutinib per the investigator’s assessment occurred, dosing with acalabrutinib will be withheld. Acalabrutinib treatment will be resumed at a lower dose only after toxicity has resolved to ≤ grade 1. There will be no dose re-escalation for acalabrutinib after recovery from toxicity, and no intra-cohort participants dose escalation will be allowed. The study will terminate early if three DLT occur for the first six participants in Phase I ( Table 4).

**Table 4.** Cohort size assessment actions.

Cohort size	Assessment	Actions
6 participants	0 DLTs	If cohort 1: proceed to the next cohort and escalate dose If cohort 2: RP2D is established as this dose
6 participants	1–2 DLTs	If cohort 1 or 2: Expand cohort to include up to 12 evaluable patients and re-evaluate
6 participants	≥ 3 DLTs	If cohort 1: Dose will be considered non-tolerated dose (NTD) No further recruitment to this cohort and dose escalation will cease If cohort 2: The RP2D will be defined as the dose in the previous cohort or further assessment may be required of an intermediate dose
12 participants	1–2 DLTs	If cohort 1: dose escalation may occur by proceeding to the next cohort, at the safety committee’s discretion If cohort 2: RP2D may be established at this dose, at the safety committee’s discretion
12 participants	≥ 3 DLTs	If cohort 1: Dose will be considered non-tolerated dose (NTD) No further recruitment to this cohort and dose escalation will cease If cohort 2: The RP2D will be defined as the dose in the previous cohort or further assessment may be required of an intermediate dose

Dose adjustments for R-CHOP components, will follow conventional dose modification schedules.

Phase II will use ORR to document the anti-tumour activity of the acalabrutinib and R-CHOP combination therapy, as well as investigating treatment safety. Safety will be assessed throughout via:

- The reporting of adverse events using the NCI CTCAE v4.03.
- Regular haematological and biochemistry evaluation
- DLT defined using the NCI CTCAE v4.03.

***Secondary outcomes***

- Pharmacokinetics of acalabrutinib, area under the curve (AUC), maximum concentration (C _max_), time to maximum concentration (T _max_), half-life (T _1/2_) and other PK parameters
- Overall response rate of the combination acalabrutinib and R-CHOP according to cell of origin
  Progression-free survival at 2 years
- Overall survival (death from any cause) at 2 years

***Tertiary outcomes***

- Btk occupancy by acalabrutinib on peripheral blood using fluorescent affinity probe assay
- Antibody-dependent cell-mediated cytotoxicity of R-CHOP when combined to acalabrutinib, post 1st R-CHOP and at day 8, 2nd cycle acalabrutinib + R-CHOP
- CD86 and CD69 expression as a function of BCR activation by flow cytometry.
- Tumour-specific DNA in plasma will be sequenced throughout treatment and clinical course
- Apply the following techniques to formalin fixed paraffin embedded (FFPE) tumour material: mutational panel, Fluorescence In Situ Hybridization (FISH) analysis, immunohistochemical analysis for dual protein expression of Myc and Bcl2 and gene expression profiling using whole transcriptome profiling

### Participant timeline

The time schedule of enrolment, interventions, assessments, and visits for participants are fully detailed in the schedule of events ( Table 2).

### Sample size

There is no formal sample size calculation given the modified 6+6 classical design. Sample size for Phase I is based upon anticipated numbers to complete schedule of dose escalation to DLT or MAD (n=6 - 24 participants). For Phase II, the sample size (n=15) permits sufficient numbers of participants to gain additional safety information and to look for exploratory signal of efficacy in biological subgroups in a pooled analysis of both stages.

### Recruitment

Patients are approached within a hospital setting and screened for eligibility by research staff to ensure all inclusion and exclusion criteria are met. Clinicians will seek informed consent to enter the trial from a patient only after the patient has received a full explanation of the trial, the patient has read the PIS and had enough time to consider taking part.

After the participant has entered the trial the clinician remains free to give alternative treatment to that specified in the protocol at any stage if he/she feels it is in the participant’s best interest, but the reasons for doing so should be recorded. In these cases, participants will be withdrawn from protocol treatment but remain within the trial for the purposes of follow-up and data analysis.

### Assignment of interventions

ACCEPT is a non-randomised open-label study, consequently there is no allocation sequence and no-one involved in the trial is blinded to the intervention.

### Data collection and management

***Plans for assessment and collection of outcomes***

Hospital research staff will enter participant data into the study eCRFs via a remote data collection tool (Medidata Rave). Only trained personnel with specific roles in the study will be granted access to the eCRFs. SCTU trial staff will regularly check the data for missing or anomalous values. Data queries will either be automatically generated within the eCRF, or manually raised with site by the SCTU team. Site staff will respond to explain or resolve the discrepancies.

***Plans to promote participant retention and complete follow-up***

ACCEPT has no specific plans for promoting retention to the trial. Participants who do not complete six cycles of treatment for reasons other than toxicity will be replaced.

***Data management***

Full details of the data management strategy for the study are available as *Extended data* ^14^.

***Confidentiality***

Participant data is pseudonymised by assigning each participant a participant identifier code, which is used to identify the participant during the study and for any participant- specific communication between SCTU and site.

### Plans for collection, laboratory evaluation and storage of biological specimens for genetic or molecular analysis in this trial/future use

***Pharmacokinetic sampling***

Acalabrutinib pharmacokinetics will be assessed in all participants by determination of serum concentrations of acalabrutinib by a fully validated assay that has already been used in human studies. The samples will be taken from participants as explained in Table 5.

**Table 5.** Schedule for pharmacokinetic sampling.

			Hours post dose ^a^					
Cycle	Day	Predose	0.5	0.75	1	2	4	6
2	1	X	X	X	X	X	X	X
	8	X	X	X	X	X	X	X
	15	X	X	X	X	X		
3	1	X						

^a^Post dose samples should be taken at the above specified times ± 5 minutes after study drug administration

***Pharmocodynamic sampling***

Acalabrutinib pharmacodynamics will be assessed in all participants by measurements of the following:

1. Evaluation of BTK occupancy in PBMCs.

PBMC will be incubated with a fluorescently-tagged analogue of acalabrutinib. Acalabrutinib and the probe bind covalently to Btk, with binding of the probe being prevented by acalabrutinib occupancy. Probe-binding will be detected by flow cytometry. PMBCs will be collected by blood sample on day 1 of cycle 2 pre-dose and at 4 hours post-dose and on day 8 of cycle 2 pre-dose, at 2 and 4 hours post dose ( Table 6).

**Table 6.** Schedule for pharmacodynamic sampling.

					Hours post dose ^a^
	Baseline	Cycle	Day	Predose	4
BTK occupancy PBMCs		2	1	X	X
		2	8	X	
ADCC		2	1	X	X
		2	8	X	X
CD86 and CD69 expression PBMCs		2	1	X	
		2	8	X	
		4	1	X	

ADCC, Antibody dependent cellular cytotoxicity; BTK, Bruton Tyrosine Kinase; PBMC, Peripheral Blood Mononuclear cells

^a^The post dose sample should be taken 4 hours ± 5 minutes after study drug administration.

2. Impact of the addition of acalabrutinib on R-CHOP mediated ADCC

Blood samples will be collected on day 1 and day 8 cycle 2 pre- dose and 4 hours post dose. A fluorescent dye release assay measuring target cell lysis, coupled with flow cytometry will be employed to measure ADCC ( Table 6).

3. BCR activation before and after R-CHOP + acalabrutinib

Blood samples for measurement of BCR activation will be collected on day 1 and day 8 cycle 2 pre-dose and on day 1 cycle 4 pre-dose. BCR activation will be determined by measurement of CD86 and CD69 expression on PBMC by flow cytometry ( Table 6).

***Translational research***

This study will prospectively validate the cell of origin (ABC versus GCB) model of DLBCL and its practicality and utility, as well as assessing the benefit/toxicity of the addition of acalabrutinib to R-CHOP.

For all participants, sufficient diagnostic material should be available to forward to a central laboratory for pathological review and gene expression profiling. FFPE lymph node biopsies should be forwarded to Haematological Malignancy Diagnostic Service (HMDS) where molecular phenotyping will be performed using a reliable array platform requiring the extraction of RNA. Central pathological review will be performed as quality check but not fed back to site unless findings untoward. There must be sufficient tumour block to test for H/E morphological check of compatibility of DLBCL diagnosis, immunophenotyping, RNA for gene expression profiling. Additional material will be used for immunohistochemical, cytogenetic and molecular genetic studies FISH, DNA for mutation, Transcription Mediated Amplification. Material will only be retained if the sample size is sufficient for removal of material without compromising its value as archived diagnostic material. This may include the generation of extended full section immunohistochemical studies, tissue microarrays and DNA extraction. The remaining FFPE block will be returned to the local pathologist.

DNA extracted from tumour material will be used to perform mutation detection on BCR pathway (e.g. Btk, PI3K, CD79b). Genetic abnormalities of BCR pathway will be correlated with clinical outcomes and the expression of BCR pathway target genes.

### Statistical methods

***Statistical methods for primary and secondary outcomes***

A full and detailed statistical analysis plan will be developed prior to the final analysis of the trial. The main features of the statistical analysis plan are:

- Participants are required to receive 6 cycles of treatment to be included in the analysis, unless the cycles are not completed due to toxicity. The safety population will include all participants who receive any component of therapy.
- Baseline characteristics will be summarised with means and standard deviations for continuous outcomes (if data is skewed medians and ranges will be presented) and frequencies and percentages for binary outcomes.
- The primary analysis will be presented in a descriptive fashion, including a summary of any DLTs experienced by participants in Phase I and summary statistics for overall response rates for participants in Phase II, summarised with frequencies and percentages.
- The safety component of the primary analysis will consist of all AEs and SAEs summarised by the CTCAE System Organ Class and term. SAEs by relatedness will also be listed.
- Kaplan-Meier survival curves will be produced for the time to event secondary outcomes of progression-free survival and overall survival.
- The final analysis will be conducted after one of the following conditions is met.
  ○ The trial is terminated early (for example, due to toxicity).
  ○ All participants have had the opportunity to complete protocol defined therapy and have completed the final follow-up visit, which will be 2 years after receiving the last study treatment, or sooner, if all participants have progressed, died or withdrawn from the study.
  ○ The predicted date for the final analysis is quarter 3 of 2022

***Interim analyses***

In Phase I the Safety Review Committee will review safety data and advise on dose escalation. The study will terminate early if three DLT occur for the first 6 participants in Phase I.

In Phase II an independent Data Monitoring & Ethics Committee (DMEC) will review outcome and safety data regularly during the trial advising the independent Trial Steering Committee (TSC) on continuation of the trial.

***Methods for additional analyses (e.g. subgroup analyses)***

Not applicable.

***Methods in analysis to handle protocol non-adherence and any statistical methods to handle missing data***

If the participant is discovered to be ineligible over the course of the study their participation will end. Where possible, participants who have withdrawn from trial treatment should remain in follow-up as per the trial schedule. If participants additionally withdraw consent for this, they should revert to standard clinical care as deemed by the responsible clinician. It would remain useful for the trial team to continue to collect survival follow-up data and unless the participant explicitly states otherwise, survival follow-up data will continue to be collected. Details of trial discontinuation (date, reason if known) should be recorded in the eCRF and medical record.

***Plans to give access to the full protocol, participant level-data and statistical code***

The full protocol is available as *Extended data* ^14^. The ‘Dissemination plans’ section of this article describes how to access trial-related data.

### Oversight and monitoring

***Composition of the coordinating centre and trial steering committee***

An independent TSC has been set up to monitor trial progress. The ACCEPT TSC charter defines the membership, terms of reference, roles, responsibilities, authority, decision-making and relationships of the TSC, including the timing of meetings, frequency and format of meetings and relationships with other trial committees.

The ACCEPT Trial Management Group includes representatives with expertise in haematology, oncology, translational science and medical statistics; as well as being supported by a Patient and Public Involvement contributor and CTU staff involved in the day-to-day running of the trial. Charters for these groups are available via accept@soton.ac.uk.

***Composition of the data monitoring committee, its role and reporting structure***

An independent DMEC comprising three clinicians and a statistician experienced in this research area (but not directly involved in this trial apart from DMEC membership) has been set up to monitor trial progress and safety. The Charter for this group is also available via accept@soton.ac.uk.

***Adverse event reporting and harms***

Data on AEs will be collected at treatment and follow-up visits. SAEs will be reported up to 30 days after the last administration of the trial drug to the SCTU safety desk. The trial also has a UK regulatory compliant real-time SAE reporting process to identify serious AEs and suspected unexpected SAEs that could suspend or stop the trial if warranted.

***Frequency and plans for auditing trial conduct***

The SCTU has undertaken a risk assessment for the ACCEPT trial, which includes the requirements for monitoring (both central and site). The SCTU undertakes a number of internal audits of its own systems and processes annually and has routine audits from both its sponsor and the independent Medicine and Health care products regulatory authority (MHRA).

***Plans for communicating important protocol amendments to relevant parties (e.g. trial participants, ethical committees)***

Research ethics committee/MHRA-approved protocol amendments will be communicated to sites via email and updated trial documentation provided centrally via the SCTU trial website ( https://www.southampton.ac.uk/ctu). Trial registries will be amended where relevant with explanations for these changes.

### End of the trial

The end of the study is defined as the date of the last follow-up visit of the last participant (expected to occur 24 months after the last participant receives their last study treatment). NB: The study will terminate early if three DLT occur for the first 6 participant in Phase I.

## Dissemination plans

The results from the ACCEPT trial will be disseminated to patients and clinical teams through peer-reviewed journal articles authored by the members of the trial management group and presented at international conferences. We will also publicise our findings through existing networks and patient groups. Summary trial results will be available on the SCTU website.

Pseudonymised individual participant data within the clinical trial dataset will be available for sharing via controlled access by authorised SCTU staff (as delegated to SCTU by the trial sponsor). Data access can be requested via a SCTU Data Release application form; detailing the specific requirements and the proposed research, statistical analysis, publication plan and evidence of research group qualifications. Please email the completed form to the SCTU Data Release Committee Coordinator at ctu@soton.ac.uk.

Data access requests are reviewed against specific eligibility criteria by the SCTU data custodian and key members of the trial team, including a statistician and chief investigator or by an external Independent Review Panel. Decisions about requests are made promptly and usually no more than three months after receipt of request. Responses to all data requests, with a clear rationale for any refusals, will be sent promptly to the data requester.

## Trial status

Recruitment opened in May 2017 and finished January 2020; the trial is now closed to recruitment and is in follow-up. However, if any of the current participants (n=9) withdraw prior to receiving 6 cycles of R-CHOP, for reasons other than toxicity, we will need to replace them. If this situation arises, we will re-open recruitment to all sites.

If the trial does not need to reopen, the last participants will reach cycle 6 on 20 April 2020 and will complete treatment on 6 July 2020. Participants enter a 24-month follow-up period after treatment and LPLV will be July 2022. The Phase I participants are nearing the end of their follow-up period and FPLV is due on 15 Feb 2020.

This clinical trial was entered into EudraCT in December 2017 (EudraCT Number: 2015-003213-18) and is registered as ISRCTN 13626902.

## Discussion

ACCEPT aims to find out the maximum dose of acalabrutinib that can safely be given to patients in combination with R-CHOP as well as investigating the potential side effects and activity in patients with DLBCL.

### First use of drug in combination with R-CHOP

This is the first use of the second generation Btk inhibitor acalabrutinib in combination with R-CHOP. This has been addressed through use of an appropriate Phase I trial design and monitoring during the study. The protocol contains stopping criteria in response to side effects which may occur with dose levels. To maintain safety during the trial, the data will be monitored closely by a Safety Review Committee and an independent DMEC on an on-going basis. First generation Btk inhibitors have been used safely in combination with R-CHOP in this patient group.

### Uncertainty

Previous preclinical and clinical observations make a compelling case for the clinical investigation of R-CHOP in combination with acalabrutinib for the treatment of patients with DLBCL. However, the clinical effects of acalabrutinib on DLCBL are unknown and the initial group of patients in particular are unlikely to derive benefit from low dose acalabrutinib. It will be made clear to all patients that the benefit of acalabrutinib is uncertain.

### Biological samples to be taken specifically for this study

Multiple blood samples will be taken in this study. Participants may also be subjected to up to two bone marrow biopsies and a lumbar puncture. These study procedures have been designed to be as limited as possible to gain the necessary information and to be conducted in the least distressing way by experienced health care professionals.

### Ionising radiation

Participants will have two PET/CT scans and a separate CT with contrast (Cycle 7). These are standard of care in DLBCL. In addition, they will undergo two additional CT scans to look for progression as a research investigation (months 12 and 24). Patients will be made fully aware of this and the risks of these scans prior to giving consent to their participation in the trial.

In addition, an Echocardiogram or MUGA will be performed for all patients to establish whether patients have a left ventricular ejection fraction of >55% to ensure they are suitable to receive 300mg/m ^2^ doxorubicin.

### Legal issues

There are no particular legal concerns arising from this study. The SCTU at the University of Southampton will be the UK coordinating trials unit and the University Hospital Southampton NHS Foundation Trust the sponsor in the UK and responsible for all legal requirements of conducting a non-commercial clinical trial of an investigation product. The drug for this trial is provided by ACERTA Pharma, BV who are responsible for the Good Manufacturing Practice quality drug and who is also providing research funding.

## Data availability

### Underlying data

No underlying data is associated with this article.

### Extended data

Figshare: ACCEPT - combining acalabrutinib with rituximab, cyclophosphamide, doxorubicin, vincristine and prednisolone (R-CHOP) for Diffuse Large B-cell Lymphoma (DLBCL): study protocol for a phase Ib/II open-label non-randomised clinical trial, https://doi.org/10.6084/m9.figshare.11791275.v4 ^14^.

This project contains the following extended data:

- Informed Consent Form and Patient Information Sheet – Tissue Block Screening; Phase I; Phase II; Pregnancy
- Full trial protocol
- Data Management Plan

Data are available under the terms of the Creative Commons Attribution 4.0 International license (CC-BY 4.0).

### Reporting guidelines

Figshare: SPIRIT checklist for ‘ACCEPT - combining acalabrutinib with rituximab, cyclophosphamide, doxorubicin, vincristine and prednisolone (R-CHOP) for Diffuse Large B-cell Lymphoma (DLBCL): study protocol for a phase Ib/II open-label non-randomised clinical trial’, https://doi.org/10.6084/m9.figshare.11791275.v4 ^14^.

Data are available under the terms of the Creative Commons Attribution 4.0 International license (CC-BY 4.0).

## Ethics approval and consent to participate

The study received ethical approval from South Central - Berkshire Research Ethics Committee on 26 January 2017 (ref: 16/SC/0657) and has Health Research Authority approval (IRAS 182046). Written informed consent for participation and publication of the participants/patients’ data was obtained from the participants. Participants are free to withdraw at any time. The Informed Consent Forms are provided as *Extended data.*

## Consent for publication

Responsibility for publication has been delegated to Andrew Davies (Chief Investigator) and Gareth Griffiths (Director of SCTU) who have consented to this publication.

## Sponsor information

Name and contact information for the trial sponsor: University Hospital Southampton NHS Foundation Trust (email: R&Doffice@suht.swest.nhs.uk), sponsor reference number: RHM CAN 1129.

The study sponsor was not involved in the study design; writing of the protocol paper; or the decision to submit the paper for publication.
