# Supplementary material for: ACCEPT - combining acalabrutinib with rituximab, cyclophosphamide, doxorubicin, vincristine and prednisolone (R-CHOP) for Diffuse Large B-cell Lymphoma (DLBCL): study protocol for a Phase Ib/II open-label non-randomised clinical trial
Source: F1000Res. 2020 Aug 7;9:941. [Version 1] doi: 10.12688/f1000research.22318.1 (PMC7551525; doi:10.12688/f1000research.22318.1)
Supplement: Peer review history

# Peer review: Reviewer response for version 1

**DOI:** 10.5256/f1000research.24618.r72306

**Published:** 2020-10-12

**Authors:** Wendy Osborne

**Affiliations:** 1 Department of Haematology, Freeman Hospital, Newcastle Upon Tyne Hospitals NHS Foundation Trust, Newcastle Upon Tyne, UK

- Rationale clear and an important unmet need to address, in view of the poor outcomes for patients with R/R DLBCL. Biological evidence already available to confirm dysregulation of BCR signalling and in agreement with rationale for a second generation Btk inhibitor.
- Table 1 is clear and of value to the manuscript.
- Figure 1 is clear and well designed, end of treatment CT is listed in this figure but standard of care 2 PET scans and a separate CT is described in ionising radiation paragraph. Imaging to be clarified on figure 1.
- Inclusion and exclusion criteria as standard for target population.
- Dose limiting toxicities are also well defined and appropriate for this combination and design.
- Translational research is clearly described and of value to the aims of the study, pathology review is performed and essential to have within this trial design. There is clear and appropriate governance structure for the trial.
- In summary, excellent rationale and design with no concerns about protocol, imaging use to be clarified on figure 1.

Is the study design appropriate for the research question?

Yes

Is the rationale for, and objectives of, the study clearly described?

Yes

Are sufficient details of the methods provided to allow replication by others?

Yes

Are the datasets clearly presented in a useable and accessible format?

Yes

Reviewer Expertise:

Clinical and biological lymphoma

I confirm that I have read this submission and believe that I have an appropriate level of expertise to confirm that it is of an acceptable scientific standard.

# Peer review: Reviewer response for version 1

**DOI:** 10.5256/f1000research.24618.r69037

**Published:** 2020-10-01

**Authors:** Allan Hackshaw

**Affiliations:** 1 Cancer Research UK Lung Cancer Centre of Excellence, University College London Cancer Institute, London, UK; 2 University College London, London, UK

A well-designed and efficient phase I then II trial. The phase I stage involves finding whether once or twice daily acalabrutinib has acceptable safety. This then rolls into a single arm phase II trial. All of the endpoints are in common use. This project also has a key translational component, making this trial more informative at the end.

Is the study design appropriate for the research question?

Yes

Is the rationale for, and objectives of, the study clearly described?

Yes

Are sufficient details of the methods provided to allow replication by others?

Yes

Are the datasets clearly presented in a useable and accessible format?

Not applicable

Reviewer Expertise:

Cancer clinical trials

I confirm that I have read this submission and believe that I have an appropriate level of expertise to confirm that it is of an acceptable scientific standard.
